# Validation List no. 220. Valid publication of new names and new combinations effectively published outside the IJSEM

**DOI:** 10.1099/ijsem.0.006501

**Published:** 2024-11-28

**Authors:** Aharon Oren, Markus Göker

**Affiliations:** 1 The Institute of Life Sciences, The Hebrew University of Jerusalem, The Edmond J. Safra Campus, 9190401 Jerusalem, Israel; 2 Leibniz Institute DSMZ – German Collection of Microorganisms and Cell Cultures, Inhoffenstrasse 7B, 38124 Braunschweig, Germany

The purpose of this list is to publish in the *International Journal of Systematic and Evolutionary Microbiology* (IJSEM) those new names and new combinations that were effectively published outside the IJSEM as set forth in Rule 27 of the International Code of Nomenclature of Prokaryotes (2022 Revision) [1]. Authors and other individuals wishing to have new names and/or combinations included in future lists should send an electronic copy of the published paper to the IJSEM Editorial Office for confirmation that all of the other requirements for valid publication have been met. It is also a requirement of IJSEM and the International Code of Nomenclature of Prokaryotes (ICNP) that authors of new species, new subspecies and new combinations provide evidence that types are deposited in two recognized culture collections in two different countries. It should be noted that the date of valid publication of these new names and combinations is the date of publication of this list, not the date of the original publication of the names and combinations. The authors of the new names and combinations are as given below.

Inclusion of a name on these lists validates the publication of the name and thereby makes it available in the nomenclature of prokaryotes. Inclusion of a name on these lists is part of the requirements of valid publication of an effectively published name. Indeed, some of these names may, in time, be considered later synonyms, or it may be proposed to transfer the organisms to another genus, thus necessitating the creation of a new combination. Conversely, it may be proposed to transfer an organism back to an earlier genus and to regard the basonym or an earlier new combination as the correct name instead of a more recent new combination. The ICNP does not decide on such taxonomic opinions.

**Table:** 

Name/authors	Proposed as	Nomenclatural type^1^	Priority^2^	Reference
*Abyssisolibacter* Kim *et al*. 2016, 351	gen. nov.	*Abyssisolibacter fermentans*	48	[2]
*Abyssisolibacter fermentans* Kim *et al*. 2016, 351	sp. nov.	MCWD3 (=JCM 31420=KCTC 15524)	48	[2]
*Acidocellaceae* Degli Esposti *et al*. 2023, 19^3,4^	fam. nov.	*Acidocella^5^*	12	[3]
*Acidovorax bellezanensis* Sánchez-Castro *et al*. 2024, 6^3^	sp. nov.	Be4 (=CECT 30865=DSM 116209)	13	[4]
*Acinetobacter oleivorans* Kang *et al*. 2011, 33^6^	sp. nov.	DR-1 (=JCM 16667=KCTC 23045)	3	[5]
*Adlercreutzia faecimuris* Suh *et al*. 2024, 6^3^	sp. nov.	JBNU-10 (=CCUG 75610=KCTC 25028)	41	[6]
*Altererythrobacter flavalbus* Liu *et al*. 2024, 8^3^	sp. nov.	HHU E2-1 (=CGMCC 1.17394=KCTC 72835=MCCC 1K04226)	37	[7]
*Anaerobaca* Khomyakova *et al*. 2024, 8^3^	gen. nov.	*Anaerobaca lacustris*	5	[8]
*Anaerobaca lacustris* Khomyakova *et al*. 2024, 8^3^	sp. nov.	M17dextr (=DSM 113417=JCM 39238=KCTC 25381=UQM 41474=VKM B-3571)	5	[8]
*Anaerobacaceae* Khomyakova *et al*. 2024, 8^3^	fam. nov.	*Anaerobaca*	5	[8]
*Asprobacillus* Rey-Velasco *et al*. 2024, 23^3^	gen. nov.	*Asprobacillus argus* corrig.	1	[9]
*Asprobacillus argus* corrig. Rey-Velasco *et al*. 2024, 23^3,7^	sp. nov.	S356 (=CCM 9388=CECT 30474)	1	[9]
*Atrimonadaceae* Kawamoto *et al*. 2024, 8^3^	fam. nov.	*Atrimonas*	27	[10]
*Atrimonas* Kawamoto *et al*. 2024, 5^3^	gen. nov.	*Atrimonas thermophila*	27	[10]
*Atrimonas thermophila* Kawamoto *et al*. 2024, 5^3^	sp. nov.	M15 (=JCM 39389=KCTC 25731)	27	[10]
*Autumnicola* Rey-Velasco *et al*. 2024, 24^3^	gen. nov.	*Autumnicola musiva*	1	[9]
*Autumnicola edwardsiae* Rey-Velasco *et al*. 2024, 25^3^	sp. nov.	F297 (=CCM 9342=CECT 30794)	1	[9]
*Autumnicola lenta* Rey-Velasco *et al*. 2024, 25^3^	sp. nov.	F260 (=CCM 9346=CECT 30806)	1	[9]
*Autumnicola musiva* Rey-Velasco *et al*. 2024, 24^3,8^	sp. nov.	F117 (=CCM 9344=CECT 30804)	1	[9]
*Autumnicola patrickiae* Rey-Velasco *et al*. 2024, 24^3^	sp. nov.	F188 (=CCM 9341=CECT 30805)	1	[9]
*Autumnicola psychrophila* Rey-Velasco *et al*. 2024, 25^3^	sp. nov.	F225 (=CCM 9347=CECT 30793)	1	[9]
*Autumnicola tepida* Rey-Velasco *et al*. 2024, 24^3^	sp. nov.	F363 (=CCM 9348=CECT 30820)	1	[9]
*Banduia* Rey-Velasco *et al*. 2024, 20^3^	gen. nov.	*Banduia mediterranea*	1	[9]
*Banduia mediterranea* Rey-Velasco *et al*. 2024, 21^3^	sp. nov.	W345 (=CCM 9345=CECT 30811)	1	[9]
*Bartonella tamiae* Kosoy *et al*. 2008, 775	sp. nov.	Th239 (=JCM 14580=NCTC 13398)^9^	14	[11]
*Benzoatithermus* Hu *et al*. 2024, 8^3^	gen. nov.	*Benzoatithermus flavus*	25	[12]
*Benzoatithermus flavus* Hu *et al*. 2024, 9^3^	sp. nov.	SYSU G07066 (=GDMCC 1.2941=KCTC 92211)	25	[12]
*Borrelia nietonii* Schwan *et al*. 2024, 282	sp. nov.	MTW-2 (=ATCC TSD-355=DSM 115472)	2	[13]
*Brevibacterium metallidurans* Manzoor *et al*. 2024, 11^3^	sp. nov.	NCCP-602 (=CGMCC 1.62055=JCM 18882)	61	[14]
*Brumicola* Rey-Velasco *et al*. 2024, 22^3^	gen. nov.	*Brumicola pallidula*	1	[9]
*Brumicola blandensis* Rey-Velasco *et al*. 2024, 22^3,10^	sp. nov.	W409 (=CCM 9336=CECT 30798)	1	[9]
*Brumicola nitratireducens* (Baik *et al*. 2006) Rey-Velasco *et al*. 2024, 22^3^	comb. nov. [basonym: *Glaciecola nitratireducens* Baik *et al*. 2006]	FR1064 (=JCM 12485=KCTC 12276)	1	[9]
*Brumicola pallidula* (Bowman *et al*. 1998) Rey-Velasco *et al*. 2024, 22^3^	comb. nov. [basonym: *Glaciecola pallidula* Bowman *et al*. 1998]	IC079 (=ATCC 700757=CIP 105819=DSM 14239)^11^	1	[9]
*Corallococcus caeni* Tomita *et al*. 2024, 7^3^	sp. nov.	KH5-1 (=JCM 36609=NCIMB 15510)	54	[15]
*Coralloluteibacterium thermophilum* corrig. You *et al*. 2018, 1586^12^	sp. nov.	B51-30 (=CGMCC 1.13574=KCTC 62780)	31	[16]
*Croceicoccus esteveae* Rey-Velasco *et al*. 2024, 20^3^	sp. nov.	F390 (=CCM 9349=CECT 30821)	1	[9]
*Croceitalea rosinachiae* Rey-Velasco *et al*. 2024, 25^3^	sp. nov.	F388 (=CCM 9338=CECT 30807)	1	[9]
*Croceitalea vernalis* Rey-Velasco *et al*. 2024, 26^3^	sp. nov.	P007 (=CCM 9330=CECT 30801)	1	[9]
*Desulfatitalea alkaliphila* Khomyakova *et al*. 2023, 6^3,13^	sp. nov.	M08but (=DSM 113909=JCM 39202=KCTC 25382=UQM 41473=VKM B-3560)	7	[17]
*Devosia rhizosphaerae* Tian *et al*. 2024, 5^3^	sp. nov.	RR2S18 (=CGMCC 1.19274=KCTC 92092)	59	[18]
*Diaphorobacter limosus* Yang *et al*. 2024, 6^3^	sp. nov.	Y-1 (=CCTCC AB 2023032=KCTC 92852)	11	[19]
*Ferviditalea* Chen *et al*. 2024, 5^3^	gen. nov.	*Ferviditalea candida*	35	[20]
*Ferviditalea candida* Chen *et al*. 2024, 5^3^	sp. nov.	SYSU GA230002 (=GDMCC 1.4160=KCTC 25726)	35	[20]
*Flavivirga abyssicola* Yang *et al*. 2024, 18	sp. nov.	MEBiC7777 (=JCM 36477=KCTC 92563)	47	[21]
*Flavivirga spongiicola* Yang *et al*. 2024, 17	sp. nov.	MEBiC05379 (=JCM 16662=KCTC 92527)^14^	47	[21]
*Flavobacterium rivulicola* Kim *et al*. 2024, 6^3^	sp. nov.	IMCC34852 (=KACC 23133=KCTC 82066=NBRC 114419)	56	[22]
*Glaciecola petra* Rey-Velasco *et al*. 2024, 21^3,15^	sp. nov.	P117 (=CCM 9332=CECT 30809)	1	[9]
*Halalkalibacter flavus* Jin *et al*. 2024, 9^3^	sp. nov.	HR 1-10 (=JCM 36285=MCCC 1K08312)^16^	51	[23]
*Halalkalibacter lacteus* Jin *et al*. 2024, 10^3^	sp. nov.	J-A-003 (=GDMCC 1.2949=JCM 36286)^17^	51	[23]
*Ignisphaera cupida* Podosokorskaya *et al*. 2024, 6^3^	sp. nov.	4213-co (=JCM 39446=VKM B-3715)^18^	53	[24]
*Jeotgalibacillus haloalkalitolerans* Zhang *et al*. 2024, 8^3,19^	sp. nov.	HH7-29 (=KCTC 43417=MCCC 1K07541)	18	[25]
*Lysinibacillus pinottii* Dunlap *et al*. 2024, 7^3,20^	sp. nov.	PB211 (=CCUG 77181=NRRL B-65672)	44	[26]
*Marinobacter albus* Deng *et al*. 2024, 7^3^	sp. nov.	M216 (=KCTC 82894=MCCC 1K08600)	10	[27]
*Marinobacter apostichopi* Kudo *et al*. 2024, 10^3^	sp. nov.	LA51 (=JCM 36175=LMG 33145)	19	[28]
*Methylobacterium nigriterrae* Chen *et al*. 2024, 7^3^	sp. nov.	SYSU BS000021 (=GDMCC 1.3814=KCTC 8051)	26	[29]
*Microbacterium aquilitoris* Lee *et al*. 2024, 5^3^	sp. nov.	KSW-18 (=KCTC 49623=NBRC 115222)	15	[30]
*Microcosmobacter* Rey-Velasco *et al*. 2024, 23^3,21^	gen. nov.	*Microcosmobacter mediterraneus*	1	[9]
*Microcosmobacter mediterraneus* Rey-Velasco *et al*. 2024, 23^3^	sp. nov.	W332 (=CCM 9339=CECT 30810)	1	[9]
*Mycobacterium barrassiae* Adékambi *et al*. 2006, 3497^22^	sp. nov.	N7 (=CCUG 50398=NBRC 113646)^23^	34	[31]
*Mycolicibacterium acapulense* Gupta *et al*. 2018, 35^3^	sp. nov.	AC-103 (=ATCC 14473=JCM 6402)	32	[32]
*Nitrospirillum viridazoti* Baldani *et al*. 2024, 10^3^	sp. nov.	BR 11140 (=ATCC 35120=DSM 2788)	60	[33]
*Novipirellula maiorica* Sreya *et al*. 2023, 260^24^	sp. nov.	SM1 (=DSM 24050=JCM 17615)	58	[34]
*Paludisphaera mucosa* Ivanova *et al*. 2023, 490	sp. nov.	Pla2 (=KCTC 92668=VKM B-3698)	52	[35]
*Paracoccus benzoatiresistens* Mu *et al*. 2024, 12^3^	sp. nov.	EF6 (=GDMCC 1.3400=JCM 35642=MCCC 1K08702)	45	[36]
*Patiriisocius hiemis* Rey-Velasco *et al*. 2024, 23^3^	sp. nov.	W242 (=CCM 9333=CECT 30795)	1	[9]
*Pedobacter punctiformis* Woo *et al*. 2024, 9^3^	sp. nov.	HCMS5-2 (=KACC 22863=TBRC 16598)	8	[37]
*Pedobacter rhodius* Woo *et al*. 2024, 7^3^	sp. nov.	SJ11 (=KACC 22884=TBRC 16597)	8	[37]
*Peloplasma* Khomyakova *et al*. 2024, 11^3^	gen. nov.	*Peloplasma aerotolerans*	6	[38]
*Peloplasma aerotolerans* Khomyakova *et al*. 2024, 11^3^	sp. nov.	M4Ah (=DSM 112561=UQM 41475=VKM B-3485)	6	[38]
*Photorhabdus africana* Machado *et al*. 2024, 8^3^	sp. nov.	CRI-LC (=CCM 9390=CCOS 2112)	21	[39]
*Pleionea litopenaei* Rho *et al*. 2024, 7^3^	sp. nov.	HL-JVS1 (=JCM 36490=KCCM 90514)	28	[40]
*Polycladidibacter* Hinger *et al*. 2020, 12^3^	gen. nov.	*Polycladidibacter hongkongensis*	49	[41]
*Polycladidibacter hongkongensis* (Xu *et al*. 2015) Hinger *et al*. 2020, 12^3^	comb. nov. [basonym: *Pseudovibrio hongkongensis* Xu *et al*. 2015]	UST20140214-015B (=KCTC 42383=MCCC 1K00451)	49	[41]
*Polycladidibacter stylochi* (Zhang *et al*. 2016) Hinger *et al*. 2020, 12^3^	comb. nov. [basonym: *Pseudovibrio stylochi* Zhang *et al*. 2016]	UST20140214-052 (=KCTC 42384=MCCC 1K00452)	49	[41]
*Pricia mediterranea* Rey-Velasco *et al*. 2024, 26^3^	sp. nov.	S334 (=CCM 9387=CECT 30471)	1	[9]
*Providencia huashanensis* Yang *et al*. 2024, 4^3,25^	sp. nov.	CRE-3FA-0001 (=CCTCC AB 2023186=KCTC 8373)	39	[42]
*Pseudoalteromonas apostichopi* Kudo *et al*. 2024, 9^3^	sp. nov.	FE4 (=JCM 36173=LMG 33143)	19	[28]
*Pseudobacillus* Verma *et al*. 2019, 368	gen. nov.	*Pseudobacillus badius*	50	[43]
*Pseudobacillus badius* (Batchelor 1919) Verma *et al*. 2019, 368	comb. nov. [basonym: *Bacillus badius* Batchelor 1919 (Approved Lists 1980)]	DSM 23 (=ATCC 14574)	50	[43]
*Pseudobacillus wudalianchiensis* (Liu *et al*. 2017) Verma *et al*. 2019, 368	comb. nov. [basonym: *Bacillus wudalianchiensis* Liu *et al*. 2017]	FJAT-27215 (=CCTCC AB 2015266=DSM 100757)	50	[43]
*Pseudodesulfovibrio pelocollis* Slobodkina *et al*. 2024, 7^3^	sp. nov.	SB368 (=DSM 111087=VKM B-3585)	29	[44]
*Pseudogemmatithrix* Haufschild *et al*. 2024, 14^3^	gen. nov.	*Pseudogemmatithrix spongiicola*	57	[45]
*Pseudogemmatithrix spongiicola* Haufschild *et al*. 2024, 14^3^	sp. nov.	Strain 318 (=CECT 9875=DSM 109487=LMG 31380=VKM B-3446)	57	[45]
*Pseudomonas aestiva* Poli *et al*. 2024, 16^3^	sp. nov.	DGS32 (=CECT 31014=DSM 117504	40	[46]
*Pseudomonas grandcourensis* Poli *et al*. 2024, 15^3,26^	sp. nov.	DGS24 (=CECT 31011=DSM 117501)	40	[46]
*Pseudomonas helvetica* Poli *et al*. 2024, 16^3,27^	sp. nov.	DGS28 (=CECT 31013=DSM 117503	40	[46]
*Pseudomonas purpurea* Poli *et al*. 2024, 16^3^	sp. nov.	DGS26 (=CECT 31012=DSM 117502)	40	[46]
*Reyranella humidisoli* Lee and Kim 2024, 457	sp. nov.	MMS21-HV4-11 (=KCTC 82780=LMG 32365)	42	[47]
*Rheinheimera faecalis* Park *et al*. 2023, 6^3^	sp. nov.	YR1 (=JCM 34823=KACC 22402)	4	[48]
*Rhinopithecimicrobium* Wang *et al*. 2024, 9^3^	gen. nov.	*Rhinopithecimicrobium faecis*	17	[49]
*Rhinopithecimicrobium faecis* Wang *et al*. 2024, 9^3^	sp. nov.	WQ 2009 (=CCTCC AB 2021153=KCTC 82941)	17	[49]
*Roseiterribacter* Nakai *et al*. 2024, 13^3^	gen. nov.	*Roseiterribacter gracilis*	16	[50]
*Roseiterribacter gracilis* Nakai *et al*. 2024, 13^3,28^	sp. nov.	TMPK1 (=JCM 34627=KCTC 82790)	16	[50]
*Roseiterribacteraceae* Nakai *et al*. 2024, 13^3,28,29^	fam. nov.	*Roseiterribacter*	16	[50]
*Roseomonadaceae* Degli Esposti *et al*. 2023, 20^3,30^	fam. nov.	*Roseomonas* ^31^	12	[3]
*Rosettibacter* Podosokorskaya *et al*. 2024, 9^3^	gen. nov.	*Rosettibacter primus*	30	[51]
*Rosettibacter firmus* Podosokorskaya *et al*. 2024, 9^3^	sp. nov.	4137-Me (=JCM 39243=KCTC 92117=VKM B-3555)^32^	30	[51]
*Rosettibacter primus* Podosokorskaya *et al*. 2024, 9^3^	sp. nov.	4148-Me (=JCM 39244=KCTC 92118=VKM B-3558)^33^	30	[51]
*Rubrivirga litoralis* Rey-Velasco *et al*. 2024, 26^3,15^	sp. nov.	F394 (=CCM 9352=CECT 30808)	1	[9]
*Serratia sarumanii* Klages *et al*. 2024, 8^3^	sp. nov.	K-M0706 (=DSM 116040=LMG 33111)	20	[52]
*Shewanella salipaludis* Park *et al*. 2020, 6^3^	sp. nov.	SHSM-M6 (=KACC 19901=NBRC 113646)	33	[53]
*Spectribacter* Rey-Velasco *et al*. 2024, 21^3,34^	gen. nov.	*Spectribacter hydrogenoxidans* corrig.	1	[9]
*Spectribacter acetivorans* Rey-Velasco *et al*. 2024, 21^3^	sp. nov.	P385 (=CCM 9351=CECT 30819)	1	[9]
*Spectribacter hydrogenoxidans* corrig. Rey-Velasco *et al*. 2024, 21^3,35^	sp. nov.	W335 (=CCM 9350=CECT 30818)	1	[9]
*Sporosarcina jeotgali* Yang *et al*. 2024, 291	sp. nov.	B2O-1 (=JCM 36032=KCTC 43506)	9	[54]
*Sporosarcina oncorhynchi* Yang *et al*. 2024, 293	sp. nov.	T2O-4 (=JCM 36031=KCTC 43489)	9	[54]
*Sporosarcina trichiuri* Yang *et al*. 2024, 294	sp. nov.	0.2-SM1T-5 (=JCM 36034=KCTC 43519)	9	[54]
*Stakelama saccharophila* Rey-Velasco *et al*. 2024, 20^3^	sp. nov.	W311 (=CCM 9337=CECT 30796)	1	[9]
*Stenotrophomonas pigmentata* Li *et al*. 2024, 10^3^	sp. nov.	610A2 (=GDMCC 1.4134=JCM 36488)	43	[55]
*Streptococcus suis* subsp. *hashimotonensis* Hasegawa *et al*. 2024, 8^3^	subsp. nov.	PAGU 2482 (=CCUG 77434=GTC 18290)	46	[56]
*Streptococcus suis* subsp. suis (Kilpper-Bälz and Schleifer 1987) Hasegawa *et al*. 2024, 8^3,36^	subsp. nov.	Henrichsen S735 (=ATCC 43764=CCUG 7984=CIP 103217=DSM 9682=LMG 14181=NCTC 10234)	46	[56]
*Streptomyces antioxidans* Ser *et al*. 2016, 11^3^	sp. nov.	MUSC 164 (=DSM 101523=MCCC 1K01590)	24	[57]
*Streptomyces vulcanius* Jia *et al*. 2015, 18^37^	sp. nov.	NEAU-C3 (=CGMCC 4.7177=DSM 42139)	23	[58]
*Terrisporobacter vanillatitrophus* Böer *et al*. 2024, 18^3,38^	sp. nov.	COM (=CCOS 2104=DSM 116160)	36	[59]
*Thalassospira aquimaris* Fu *et al*. 2024, 9^3^	sp. nov.	FZY0004 (=JCM 35895=MCCC 1K08380)	38	[60]
*Thalassotalea castellviae* Rey-Velasco *et al*. 2024, 22^3^	sp. nov.	W431 (=CCM 9335=CECT 30799)	1	[9]
*Thermatribacter* Jiao *et al*. 2024, 14^3^	gen. nov.	*Thermatribacter velox*	22	[61]
*Thermatribacter velox* Jiao *et al*. 2024, 14^3^	sp. nov.	B11 (=CCAM 969=JCM 39351)	22	[61]
*Thermatribacteraceae* Jiao *et al*. 2024, 14^3^	fam. nov.	*Thermatribacter*	22	[61]
*Tropicimonas omnivorans* Rey-Velasco *et al*. 2024, 20^3,39^	sp. nov.	F158 (=CCM 9343=CECT 30792)	1	[9]
*Urechidicola vernalis* Rey-Velasco *et al*. 2024, 23^3,40^	sp. nov.	P050 (=CCM 9340=CECT 30802)	1	[9]
*Vibrio apostichopi* Kudo *et al*. 2024, 10^3^	sp. nov.	FE10 (=JCM 36174=LMG 33144)	19	[28]
*Winogradskyella marincola* Fu *et al*. 2024, 9^3^	sp. nov.	YYF002 (=JCM 35950=MCCC 1K08382)	38	[60]
*Zwartia panacis* Park *et al*. 2023, 10^3^	sp. nov.	DCY105 (=CCTCC AB 215193=KCTC 42741)	55	[62]
*Zwartia vadi* Park *et al*. 2023, 9^3^	sp. nov.	IMCC34845 (=KCTC 92920=NBRC 114902)	55	[62]

For references to Validation Lists 1–71, see *Int J Syst Bacteriol* **49** (1999) 1325. Lists 72–197 were published in *Int J Syst Evol Microbiol* **50** (2000) 3, 423, 949, 1415, 1699, 1953; and **51** (2001) 1, 263, 793, 1229, 1619, 1945; and **52** (2002) 3, 685, 1075, 1437, 1915; and **53** (2003) 1, 373, 627, 935, 1219, 1701; and **54** (2004) 1, 307, 631, 1005, 1425, 1909; and **55** (2005) 1, 547, 983, 1395, 1743, 2235; and **56** (2006) 1, 499, 925, 1459, 2025, 2507; and **57** (2007) 1, 433, 893, 1371, 1933, 2449; and **58** (2008) 1, 529, 1057, 1511, 1993, 2471; and **59** (2009) 1, 451, 923, 1555, 2129, 2647; and **60** (2010) 1, 469, 1009, 1477, 1985, 2509; **61** (2011) 1, 475,1011, 1499, 2025, 2563; and **62** (2012) 1, 473, 1017, 1443, 2045, 2549; and **63** (2013) 1, 797, 1577, 2365, 3131, 3931; and **64** (2014) 1, 693, 1455, 2184, 3603; and **65** (2015), 1, 741, 1112, 2017, 2777, 3767; and **66** (2016) 1, 1603, 1913, 2463, 3761, 4299; and **67** (2017) 1, 529, 1095, 2075, 3140, 4291; and **68** (2018), 1, 693, 1411, 2130, 2707, 3379; and **69** (2019), 5, 597, 1247, 1844, 2627, 3313, and **70** (2020) 1, 1443, 2960, 4043, 4844, 5596. Lists 197–219 were published in *Int J Syst Evol Microbiol* **71** (2021) 004600, 004688, 004773, 004846, 004943, 005096; and **72** (2022) 005167, 005260, 005331, 005422, 005517, 005592; and **73** (2023) 005709, 005812, 005845, 005931, 005997, 006080; and **74** (2024) 006173, 006229, 006275, 006398, 006452.

1 Abbreviations of culture collections cited in this list can be found at https://lpsn.dsmz.de/text/collections

2 Priority number assigned according to the date the documentation and request for validation are received.

3 The journal in which the name was effective published does not have continuous pages.

4 The preferred syllabification is A.ci.do.cel.la.ce’ae.

5 The authors also stated type species *Acidocella facilis*, but a family does not have a type species.

6 The preferred etymology is L. neut. n. *oleum*, … N.L. part. adj. *oleivorans*, …

7 The list editors have corrected the epithet to *argus* (ar’gus. Gr. masc. adj. *argos*, slow; N.L. masc. adj. *argus*, slow …).

8 The translation of L. fem. adj. *musiva* is ‘artistic’ and not ‘mosaic’.

9 The authors used the term ‘prototype strain’ instead of ‘type strain’. The effective publication states that the type strain was also deposited as ATCC BAA-1343, but no documentation was supplied.

10 The etymology should state N.L. fem. adj. instead of N.L. masc./fem. adj.

11 The effective publication states that the type strain was also deposited as ACAM 615, but no documentation was supplied.

12 The list editors have corrected the epithet to *thermophilum* (ther.mo’phi.lum. Gr. masc. adj. *thermos*, hot; N.L. masc. adj. *philus* (from Gr. masc. adj. *philos*), loving; N.L. neut. adj. *thermophilum*, heat-loving).

13 The preferred etymology is N.L. n. *alkali* … N.L. masc. adj. *philus* (from Gr. masc. adj. *philos*), …

14 The effective publication states that the type strain was also deposited as KCCM 92563, but no documentation was supplied.

15 The etymology should state L. fem. adj. instead of L. masc./fem. adj.

16 The effective publication states that the type strain was also deposited as GDMCC 1.2946, but no documentation was supplied.

17 The effective publication states that the type strain was also deposited as MCCC 1K08417, but no documentation was supplied.

18 The effective publication states that the type strain was also deposited as UQM 41593, but no documentation was supplied.

19 The preferred syllabification is ha.lo.al.ka.li.to’le.rans.

20 The etymology should state N.L. gen. n. instead of N.L. gen. masc.

21 The etymology should state Gr. masc. n. *kosmos*, … and not N.L. masc. n. Gr. masc. n.

22 The preferred syllabification is bar.ras’si.ae.

23 The effective publication states that the type strain was also deposited as CIP 108545, but no documentation was supplied.

24 The authors gave Frank 2011 as the nomenclatural authority.

25 The protologue heading should state *Providencia* instead of *P*.

26 The etymology should state N.L. fem. adj. instead of N.L. neut. adj.

27 The etymology should state L. fem. adj. instead of N.L. fem. adj.

28 The protologue heading should state nov. instead of Nov.

29 The etymology should state N.L. masc. n. *Roseiterribacter* instead of N.L. neut. n.

30 The preferred syllabification is Ro.se.o.mo.na.da.ce’ae.

31 The authors also stated type species *Roseomonas gilardii*, but a family does not have a type species.

32 The effective publication states that the type strain was also deposited as UQM 41465, but no documentation was supplied.

33 The effective publication states that the type strain was also deposited as UQM 41466, but no documentation was supplied.

34 The etymology should state N.L. masc. n. *Spectribacter*, …

35 The list editors have corrected the epithet *hydrogenooxidans* to *hydrogenoxidans* (hy.dro.gen.o’xi.dans. … N.L. part. adj. *hydrogenoxidans*, …).

36 The list editors have corrected the nomenclatural authority.

37 The etymology should state L. masc. adj. *vulcanius* instead of L. adj.

38 The etymology should state Gr. masc. adj. and N.L. masc. adj. instead of Gr. masc. Adj. and N.L. masc. Adj.

39 The preferred etymology is L. masc. adj. *omnis*, …

40 The etymology should state L. masc. adj. instead of L. fem. adj.

## Correction to Validation List no. 218 [63]

*Oceanoferula flava* corrig. Ye *et al*. 2023 should be corrected to *Oceaniferula flava* corrig. Ye *et al*. 2023.

We thank S. Turner (NCBI, NIH, Bethesda, MD, USA) for alerting us to this error.
